# Unexpected False-positive I-131 Uptake in Patients with Differentiated Thyroid Carcinoma

**DOI:** 10.4274/mirt.37450

**Published:** 2018-10-09

**Authors:** Aylin Oral, Bülent Yazıcı, Cenk Eraslan, Zeynep Burak

**Affiliations:** 1 Ege University Faculty of Medicine, Department of Nuclear Medicine, İzmir, Turkey; 2 Ege University Faculty of Medicine, Department of Radiology, İzmir, Turkey

**Keywords:** I-131, radioiodine, scintigraphy, SPECT/CT, thyroid, cancer

## Abstract

**Objective:**

Radioiodine is the most specific radionuclide for differentiated thyroid carcinoma (DTC) imaging. Despite its high specificity and sensitivity, false-positive I-131 uptake could be seen on whole body scan (WBS) that may lead to misdiagnosis and unnecessary radioiodine treatment. In this study, we aimed to present the I-131 WBS and concomitant single photon emission computed tomography/computed tomography (SPECT/CT) images of unexpected false-positive radioiodine uptake along with the patients’ clinical outcomes and the contribution of SPECT/CT imaging.

**Methods:**

I-131 WBSs of 1507 patients with DTC were retrospectively reviewed, and anticipated I-131 uptakes (like in breasts or thymus) were excluded from the study. The unexpected false-positive I-131 uptakes with concomitant SPECT/CT imaging were included in the study.

**Results:**

Twenty-one patients had 23 unexpected I-131 uptakes on WBS and concomitant SPECT/CT imaging. The vast majority (87%) of these cases were seen on post-therapeutic I-131 WBS. Most of the false-positive I-131 uptakes could be explained by SPECT/CT and radiologic findings, and were secondary to non-thyroid conditions (bronchiectasis, lung infection, subcutaneous injection into gluteal fatty tissue, aortic calcification, benign bone cyst, vertebral hemangioma, recent non-thyroid surgical procedure site, rotator cuff injury, mature cystic teratoma and ovarian follicle cyst). However, the possible reasons of 9 false-positive I-131 uptakes could not be explained by radiologic findings.

**Conclusion:**

We suggest that false-positive I-131 uptake and its underlying mechanisms (inflammation, trapping, increased perfusion, etc.) must be kept in mind in patients with thyroid cancer and unexpected findings must be considered together with serum thyroglobulin levels, SPECT/CT and radiologic findings in order to avoid misdiagnosis and unnecessary radioiodine treatment.

## Abstract (tr)

**Amaç:**

Radyoaktif iyot diferansiye tiroid karsinomu (DTK) görüntülemesinde kullanılan en yüksek özgüllüğe sahip radyonükliddir. Duyarlılık ve özgüllüğü yüksek olmakla birlikte I-131 tüm vücut tarama sintigrafisinde (TVTS) yanlış pozitif I-131 tutulumu görülebilmekte ve bu durum tanısal güçlüklere ve gereksiz tedavi uygulanmasına neden olabilmektedir. Bu çalışmada I-131 TVTS’de izlenen beklenmedik yanlış pozitif I-131 tutulumlarına ait TVTS ve tek foton emisyon bilgisayarlı tomografisi/bilgisayarlı tomografi (SPECT/BT) görüntüleme bulguları ile hastaların klinik sonuçlarının ve SPECT/BT görüntülemenin katkısının sunulması amaçlanmıştır.

**Yöntem:**

Kliniğimizde takipli 1507 DTK tanılı hastaya ait I-131 TVTS’leri retrospektif olarak incelendi ve alışılagelmiş yanlış pozitif I-131 tutulumları (timüs, meme gibi) çalışma dışında bırakıldı. Eş zamanlı SPECT/BT görüntüleri olan ve beklenen alanlar dışında izlenen yanlış pozitif I-131 tutulumları çalışmaya dahil edildi.

**Bulgular:**

Yirmi bir hastada 23 adet eş zamanlı SPECT/BT ile lokalize edilen, beklenmedik yanlış pozitif I-131 tutulumu saptandı. Bulguların büyük çoğunluğu (%87) post-terapötik TVTS’de izlendi. Yanlış pozitif tutulumların çoğunun eş zamanlı SPECT/BT ve radyolojik bulgular ile tiroid dışı nedenlere (bronşektazi, akciğer enfeksiyonu, gluteal yağlı dokuya subkutan enjeksiyon, aort kalsifikasyonu, benign kemik kisti, vertebral hemanjiom, yakın zamanlı tiroid dışı cerrahi girişim, rotator kılıf yaralanması, matür kistik teratom ve overde follikül kisti) bağlı olduğu anlaşıldı. Dokuz hastada ise yanlış pozitif iyot tutulumu radyolojik görüntüleme bulguları ile açıklanamadı.

**Sonuç:**

Tiroid kanserli hastalarda yanlış pozitif I-131 tutulumunun izlenebileceği ve altta yatan mekanizmaları (enflamasyon, kanlanma artışı, kistik lezyonda tutulum vb.) akılda tutularak, beklenmeyen I-131 tutulumlarının serum tiroglobulin düzeyi, SPECT/BT ve radyolojik verilerle birlikte değerlendirilerek gereksiz tedavilerin önüne geçilmesi gerektiği sonucuna varılmıştır.

## Introduction

Differentiated thyroid carcinoma (DTC), including papillary and follicular thyroid cancer, represents over 90% of all thyroid cancer cases (1). The primary treatment of choice in DTC is surgery (1,2). DTC patients, except microcarcinomas with no extension beyond the thyroid capsule and without lymph node or distant metastasis, receive radioiodine treatment after surgery (3). Radioiodine is the most specific (>90%) radionuclide for DTC imaging (3). Post-therapeutic I-131 whole body scan (WBS) is used for restaging while diagnostic I-131 WBS is used for the follow-up of DTC patients. Despite its high specificity and sensitivity, false-positive I-131 uptake could be seen on I-131 WBS (4,5,6,7,8,9,10). Functional (residual or metastatic) thyroid tissue is not the only tissue that accumulates radioiodine, but also salivary glands, liver, breasts and thymus could accumulate radioiodine. Also, gastrointestinal and urinary system can be visualized in radioiodine scans due to iodine excretion. In addition to these organs and systems, unexpected and false-positive radioiodine accumulation could be seen on I-131 WBS which might lead to misdiagnosis and unnecessary radioiodine treatment (8,9). Further imaging modalities are usually required to explain the unexpected I-131 uptake, but it is difficult to guide further examinations due to the absence of anatomical location data on planar imaging protocols. In such cases, single photon emission computed tomography/computed tomography (SPECT/CT) hybrid imaging is a very useful modality to determine the exact anatomic localization of the I-131 avid foci that was detected on I-131 WBS. CT component of the hybrid imaging not only improves attenuation correction, but also improves the planar data interpretation by offering the opportunity to differentiate between abnormal and physiologic structures, and sometimes low dose CT images help to diagnose the underlying pathology.

In this study, we aimed to present the imaging findings [I-131 WBS, SPECT/CT, magnetic resonance imaging (MRI), etc.], and clinical outcomes of patients as well as the contribution of SPECT/CT imaging in unexpected false-positive I-131 accumulation, and to discuss the underlying etiology of these cases.

## Materials and Methods

### Patients

From May 2012 to April 2015, 1507 DTC patients’ radioiodine scans were retrospectively reviewed. Radioiodine contaminations and expected physiologic I-131 uptakes like in the breasts or thymus were excluded from the study. Concomitant SPECT/CT imaging was performed to determine the exact anatomical localization of the I-131 avid foci on WBS and to exclude contaminations and expected physiologic I-131 uptakes. The unexpected I-131 uptakes on WBS were determined as false-positive for DTC; if non-thyroid pathologies were demonstrated by SPECT/CT and/or radiologic imaging, or no anatomic pathologies were detected by concomitant SPECT/CT or further radiologic imagings with low serum thyroglobulin (Tg) levels and negative follow-up diagnostic I-131 scans. According to these parameters, 21 patients with 23 unexpected false-positive I-13 uptakes were included in the study.

### Follow-up Protocol

In order to prevent thyroid remnants from stunning, diagnostic I-131 WBSs were not performed before the administration of therapeutic dose. Radioiodine therapy was performed according to the guideline for radioiodine therapy of DTC (11).  In the presence of abnormal findings on post-therapeutic or diagnostic I-131 WBS, concomitant SPECT/CT imaging was performed. Afterwards, further diagnostic investigations were performed in patients with abnormal laboratory or imaging findings and in patients with persistent disease, repetitive radioiodine therapies were administered at least 3 months after I-131 WBS.

### Imaging Protocol

Planar I-131 WBS was performed in both anterior and posterior projections using dual-head gamma-camera (Infinia Hawkeye 4^®^, GE Healthcare) with high-energy, parallel-hole collimators. Continuous acquisition mode was used at a table speed of 8 cm/min with a 1,024x256 matrix. The photopeak was 364 keV with a ±10% window. Additional images were required in case of unexpected iodine uptakes or accumulations that give an impression of physiologic uptake (scanning after drinking a glass of water to wash out physiologic uptake in the esophagus) or contamination (scanning after removing the contamination and taking off the stained clothes). Additional spot views are performed using a 256x256 matrix for 5 min/view.

Imaging with SPECT/CT requires a long scanning time. Therefore, in our department SPECT/CT (Infinia Hawkeye 4^®^, GE Healthcare) is not performed routinely. SPECT/CT imaging is performed for specific sites which are determined by a nuclear medicine physician based on the WBS and additional spot images. Emission SPECT images are acquired with a matrix size of 128x128, and the photopeak was 364 keV with a ±10% window. A total image of 60 frames is acquired over 360˚ with an acquisition time of 40 s/frame, angular step of 6, and zooming factor of 1. After SPECT acquisition, a CT scan is acquired for attenuation correction with a low-dose, 4-slice helical CT scanner. The CT parameters are 140 kV and 2.5 mAs. The images are reconstructed with conventional iterative algorithm, ordered subset expectation maximization and fused with CT images by using software (Xeleris^®^, GE Healthcare) for multiplanar reformatted image display.

## Results

Twenty-one patients with 23 unexpected false-positive I-131 uptakes were reviewed. The vast majority (87%) of unexpected findings were seen in post-therapeutic I-131 WBS after first ablation treatment, while the rest (13%) were seen on diagnostic WBSs.

The study included 21 patients with a median age of 58 (range 28-77 years) and a female/male ratio of 2.5:1. Nineteen patients had papillary thyroid carcinoma while 2 patients had follicular thyroid carcinoma. The histologic subtypes of papillary thyroid carcinoma were conventional in 10 (48%), follicular variant in 8 (38%) patients and 1 (5%) patient had oncocytic variant of papillary thyroid carcinoma.

Out of the 23 unexpected findings, the number of lesions located in the cranial, thoracic, abdominal and pelvic regions were 1, 15, 1 and 6, respectively. One of the patients had false-positive I-131 uptakes in both the thoracic and pelvic regions. Also, one patient had 2 false-positive uptakes in the thoracic region, one of them in the lung and the other on the rib. The locations and possible etiologies of unexpected false-positive iodine uptakes are listed in Table 1

Evaluation of the unexpected uptakes in the thoracic region (n=15), which was the most common region of false-positive I-131 uptakes in our study, revealed that 14 of them were focal uptakes while one was a mild and diffuse uptake like a thick band in the lung. On SPECT/CT images, 5, 2, 2, 1 and 5 of the false-positive uptakes in the thoracic region were located in the lung parenchyma, vascular structures, inflamed soft tissue, anterior mediastinum and bones, respectively. 

**Figure 1:**
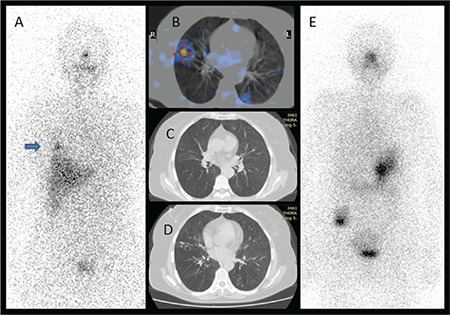
There was I-131 uptake at the lower zone of the right hemithorax on the anterior image of post-therapeutic I-131 whole body scan (A, arrow). The focal activity accumulation in the lung parenchyma on fused single photon emission computed tomography/computed tomography (CT) image (B), and findings on CT images (C, D) were concordant with bronchiectasis. The diagnostic I-131 scan (E) did not reveal any pathologic uptake and the serum thyroglobulin level was low (<0.2 ng/mL)

**Figure 2:**
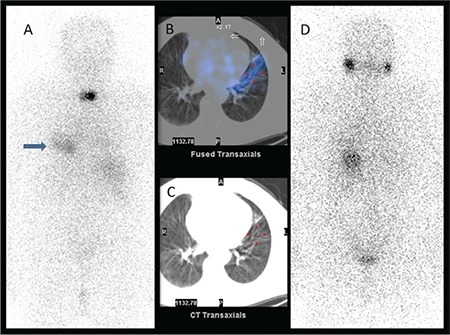
A mild diffuse I-131 uptake at the lower zone of the left hemithorax (arrow) was seen on posterior image of post-therapeutic I-131 whole body scan (A) in addition to residual thyroid tissue. The uptake in the left lung parenchyma was shaped like a thick band that could be compatible with the trajectory of a previous lung infection that was detected on fused single photon emission computed tomography/ computed tomography images (B), and no pathologic finding was found on this area on computed tomography images (C). On diagnostic I-131 scan (D) there wasn’t any pathologic uptake and the serum thyroglobulin level was low (0.7 ng/mL)

Serum Tg levels of the patients with unexpected I-131 uptakes in the lung parenchyma were low (0.3-19 ng/mL) in terms of lung metastasis. In two patients with focal activity accumulation in the lung parenchyma, it was remarkable that on CT images there were findings concordant with bronchiectasis on the same area of I-131 uptake on SPECT/CT images (Figure 1). In 2 cases, no pathology was detected that could explain the I-131 uptake in the lungs. In one case, while there was a mild and diffuse I-131 uptake that was shaped like a thick band on the left lung in SPECT/CT images, no pathologic finding was found on this area on CT images (Figure 2). On inquiry, the patient had a history of using antibiotics due to lung infection approximately one month ago. The radioiodine uptake was thought to be secondary to the previous lung infection.

The concomitant SPECT/CT imaging of five of the unexpected I-131 uptakes in the thoracic region demonstrated that I-131 involvements were localized to the bone. Serum Tg levels of these patients were low (0.3-11.9 ng/mL) for bone metastasis. In 4 cases, the activity accumulations were located to the ribs. However, no etiology that could cause I-131 involvement was deterrmined. Focal I-131 uptake of one case was located to the clavicle. This patient has been reported as a case-report earlier, in whom the focal I-131 uptake corresponded to a hypodense area in the left clavicle on CT images (4). An MRI revealed that the finding belonged to a benign lesion, a simple bone cyst.

**Figure 3:**
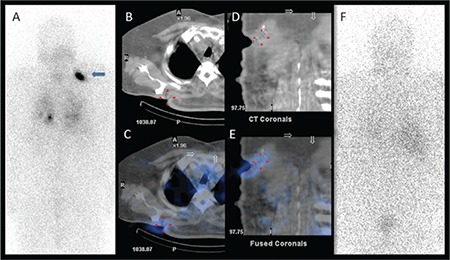
An intense I-131 uptake was identified at the upper zone of the right hemithorax (arrow) on posterior image of post-therapeutic I-131 whole body scan (A) in addition to lung metastasis. On axial computed tomography (CT) (B) and fused single photon emission computed tomography (SPECT)/CT images (C), the finding was adjacent to the right scapula, and localized to the skin/subcutaneous soft tissue. In addition, on coronal CT (D) and SPECT/CT images (E) surgical sutures related to a non-thyroidal soft tissue excision prior to radioiodine treatment were detected. On follow-up I-131 scan (F) there wasn’t any pathologic uptake but the patient still had a mildly elevated serum thyroglobulin level (7.5 ng/mL) with thyroid-stimulating hormone stimulation

One patient was treated with high dose I-131 because of multiple lung metastases with a serum Tg level of 279 ng/mL. On post-therapeutic I-131 WBS, besides the lung metastases, an intense I-131 uptake was determined at the posterior upper zone of the right hemi-thorax (Figure 3). SPECT/CT images demonstrated that the uptake was adjacent to the right scapula, and localized to skin/subcutaneous soft tissue. Moreover, surgical sutures were present in this area. It was understood that the patient had an operation due to a soft tissue lesion adjacent to the scapula before radioactive iodine (RAI) treatment, the pathology report of that lesion was interpreted as spindle-cell mesenchymal tumor. Following the second RAI treatment, the patient had no pathologic findings on I-131 WBSs and no clinical complaints. At the end of 3-years of follow-up period, the patient still has a mildly elevated serum Tg level (7.5 ng/mL) with thyroid-stimulating hormone stimulation.

Serum Tg levels of other 4 unexpected focal I-131 uptakes in the thoracic region were between 0.2 and 8 ng/mL. On SPECT/CT images, 2 of these 4 patients had focal I-131 uptake that was in accordance with aortic wall calcification and one patient had focal I-131 uptake in the anterior mediastinum without any density change on CT images. The other patient had an I-131 accumulation on the left shoulder on planar WBS that was adjacent to the left humeral head on SPECT/CT images. On inquiry, it was learned that the patient had a history of rotator cuff tear of the left shoulder. It was thought that the I-131 uptake was secondary to this condition.

When the unexpected uptakes in the pelvic region (n=6), which was the second most common region of false-positive I-131 uptakes in our study, were further evaluated, it was remarkable that all unexpected findings were identified on post-therapeutic scans with low serum Tg levels (0.2-4.6 ng/dL).

**Figure 4:**
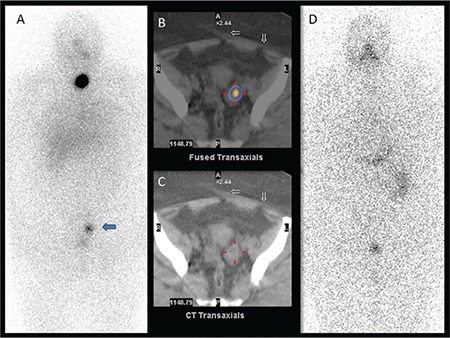
On anterior image of post-therapeutic I-131 whole body scan (A), in addition to residual thyroid tissue, there was a focal I-131 accumulation over the left side of the bladder (arrow). Fused single photon emission computed tomography/computed tomography (CT) image (B) showed that the I-131 uptake was localized to the left ovary and a hypodense cystic area in the left ovary was identified on CT image (C). The followup diagnostic I-131 scan (D) was normal and the thyroglobulin level was undetectable (<0.2 ng/mL)

On SPECT/CT images, the I-131 uptakes of 2 patients correlated to the ovaries. One of these patients was operated due to an ovarian-origin lesion and was diagnosed with mature cystic teratoma without thyroid tissue. This patient has been presented as a case report earlier (5). In the other case with I-131 uptake in the ovary, an ovarian hypo-dense cystic area was monitored on SPECT/CT images (Figure 4). Pelvic ultrasound (USG) demonstrated no pathology except a follicle cyst and it was thought that the I-131 uptake in the ovary was secondary to this cystic lesion.

**Figure 5:**
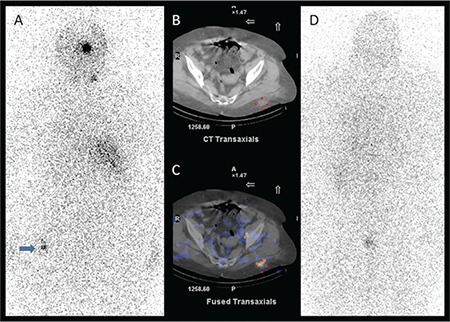
On posterior image of post-therapeutic I-131 whole body scan (A), in addition to residual thyroid tissue, there was a mild focal I-131 accumulation in the pelvic region (arrow). On axial computed tomography (CT) (B) and fused single photon emission computed tomography (SPECT)/CT (C) images, it was clear that the activity was located in the posterior gluteal soft tissue and there were density changes in the fatty tissue due to previous subcutaneous injections. The follow-up diagnostic I-131 scan (D) was normal and the thyroglobulin level was undetectable (<0.2 ng/mL)

Focal activity accumulation was observed in the pelvic region in 3 patients on posterior image of post-therapeutic I-131 WBS (Figure 5). It was hard to distinguish those activities from urinary contamination, so additional planar spot images were obtained. If the pelvic uptakes were persistent then concomitant SPECT/CT imaging was performed. On SPECT/CT images, it was found that the uptakes were localized to gluteal adipose tissue and it was evident that they matched with old injection sites in subcutaneous fatty tissue on CT images. On inquiry, all 3 cases had a history of gluteal injections within the last 2 months. Therefore, no further examination was required for these patients.

On post-therapeutic I-131 WBS, a patient had focal I-131 uptakes in the left hemithorax and posterior pelvic region. The patient’s serum Tg level was low (3.7 ng/dL) for metastasis. The thoracic radioiodine uptake corresponded to the rib, and no pathologic finding was found on CT images. The uptake in the posterior pelvic region corresponded to the fifth lumbar vertebra on SPECT/CT. On MRI images there was a hyper-intense lesion on T1 and T2-weighted sequences in accordance with hemangioma in the fifth lumbar vertebra.

In a patient with a focal I-131 uptake on the right abdominal region, the uptake was found to correlate with soft tissue at the intercostal area adjacent to the liver on SPECT/CT images. However, no etiology that can explain I-131 uptake could be detected by abdominal CT or USG.

During diagnostic I-131 WBS of one patient, focal activity accumulation was identified in the cranium on posterior planar image. On SPECT/CT, an activity was detected at the right parieto-occipital area. There wasn’t any density change on CT images. No etiology could be found by 2 cranial MRs obtained with an interval of 6 months.

Tg values were determined during follow-up diagnostic I-131 WBS in all cases, and varied between <0.2 and 1.9 ng/mL except the case with lung metastasis (stimulated Tg: 7.5 ng/mL, unstimulated Tg <0.2 ng/mL). There was no pathologic finding on diagnostic I-131 WBSs, and patients are being followed-up for 1-3 years as disease-free. All of the patients’ unstimulated Tg levels remain low (0.2 ng/mL).

## Discussion

Sodium iodide symporter expression is one of the well-known mechanisms that is responsible for RAI uptake in tissues. The physiologic I-131 uptake in the thymus, breast, salivary gland and gastrointestinal system in addition to the thyroid tissue is explained with this mechanism. In addition, false-positive uptake might be encountered in I-131 WBSs by mechanisms such as metabolism of I-131-labeled thyroid hormones (liver uptake), retention and contamination of physiologic secretions and body fluids containing radioiodine (saliva, tears, sweat, urine, blood, exudate, transudate, gastric and mucosal secretions, etc.), uptake and retention of radioiodine in inflamed tissues. Nevertheless, mechanism of the uptake of activity observed in a group of patients is not completely understood yet (8,9).

Increased perfusion and vasodilation, and enhanced capillary permeability in pulmonary infections are suggested to cause I-131 accumulation (8). Although rare, false-positive I-131 uptake secondary to active or inactive lung infections has been reported in the literature (12,13). Also, in this study, an accumulation of activity that was attributed to infection was observed in a patient with a previous history of lung infection.

Accumulation of bronchial secretions in bronchiectasis causes I-131 uptake (8). Focal I-131 accumulations that might be confused with lung metastasis due to bronchiectasis had been reported in the literature (14,15). Also, observation of activity accumulation in the bronchiectasis area in 2 patients in this study supports this finding.

To identify the exact localization of an uptake site on planar I-131 WBS is difficult due to the lack of anatomic landmarks. SPECT/CT, the hybrid imaging modality that combines SPECT scan with CT scan, is very useful to determine the exact anatomic location of the I-131 avid foci that is detected on I-131 WBS. Further imaging modalities are usually required to explain unexpected I-131 uptakes. CT component of the hybrid imaging improves the planar data interpretation by offering the opportunity to differentiate between abnormal and physiologic structures. Sometimes low dose CT component of SPECT/CT imaging helps to diagnose the underlying pathology. Maruoka et al. (16) reported that the interpretation was altered to be physiologic or benign uptake in 38% of patients with the addition of SPECT/CT. Also SPECT/CT imaging helps for choosing the optimal imaging modality (USG, MRI, contrast enhanced CT, etc.) if the CT component fails to determine the underlying etiology of an uptake site.

Three patients with I-131 uptake in the gluteal fatty tissue had a recent history of gluteal injection. This finding was thought to be secondary to the probable inflammation due to injection into the fatty tissue instead of the intramuscular area. False-positive I-131 uptake has been reported in the literature in the gluteal fatty tissue secondary to a granuloma due to foreign material (17). 

Post-therapeutic imaging of the patient with a history of partial rotator cuff tear, and of the patient who received RAI following a previously performed surgery for a skin lesion suggested that transudate and inflammation that were produced due to tissue injury might be the possible etiology of the false-positive radioiodine uptake.

The etiology of focal I-131 uptake in the area of an aortic wall calcification is not entirely known.  A false-positive uptake of I-131 has been reported in a case with aortic aneurysm in the literature (18). The aorta diameter was normal in the case presented herein, and this finding was thought to be secondary to atherosclerosis and a possible inflamed plaque corresponding to this area.

The incidence of vertebral hemangiomas is reported as approximately 11%, more frequently in the thoracic vertebrae in autopsy series. In this present study, the I-131 uptake in the lumbar vertebrae on SPECT/CT images in one case was compatible with a hemangioma as detected by MRI. The I-131 uptake in hemangiomas is attributed to intravascular blood pooling and enhanced capillary permeability. A thoracic focal activity uptake in the posterior planar image secondary to a hemangioma has been previously reported in the literature (19).

In our study, ovarian I-131 accumulation was observed in 2 patients. One of them was diagnosed with mature cystic teratoma and has been previously reported as a case report (5). The other patient had no pathology except an ovarian follicle cyst. Functional follicle cysts have been reported to demonstrate I-131 accumulation in the literature (20). Also, there are several studies published in the literature reporting false-positive I-131 accumulation in cystic structures (7,8,21,22). Radioiodine enters cysts by passive diffusion and is trapped in the cyst (8). In this paper, a case with I-131 accumulation due to a benign bone cyst in the clavicle is also included, who has been previously reported (4).

In this study, the possible etiology of 9 false-positive I-131 uptakes in 8 patients remained unclear. I-131 uptakes in the remaining patients were mostly associated with inflammation. The radioiodine uptakes in the undetermined group were thought to be secondary to inflammation that could not be demonstrated by radiologic findings. This might be explained by recovery of the possible temporary and mild inflammation within the time period between radioiodine uptake and further radiologic examination (<4 weeks).

Evaluation of false-positive uptake in the neck area is important since it may be confused with residual thyroid tissue or metastatic lymph nodes. However, the contribution of adequate patient history (presence of metastatic disease, serum Tg values, findings of previous imaging studies and etc.) obtained from the clinician is very helpful on the evaluation of I-131 WBSs. SPECT/CT examination has significant importance to prevent unnecessary examinations and treatment, if available for the evaluation of unexpected radioiodine uptake. 

The importance of prevention of unnecessary treatments has also been emphasized in the literature, by taking false-positive uptake rates and laboratory findings (Tg), clinical and imaging data into consideration in addition to I-131 WBS findings of a particular patient (6,14,23).

## Conclusion

Unexpected radioiodine uptake secondary to various extra-thyroidal reasons (inflammation, increased blood supply, trapping in the cystic lesion, etc.) should be kept in mind while interpreting I-131 WBSs, especially in post-therapeutic scans due to the higher dose applied. In patients with notably discordant clinical and laboratory data, accurate localization of radioiodine uptake is important. At this point, SPECT/CT imaging is the method of choice to both localize the unexpected foci and aid differential diagnosis. It is concluded that the unexpected finding should be enlightened by using different imaging models (MRI, USG, etc.) according to the specifications of the tissue thus preventing unnecessary treatment.

## Figures and Tables

**Table 1:**
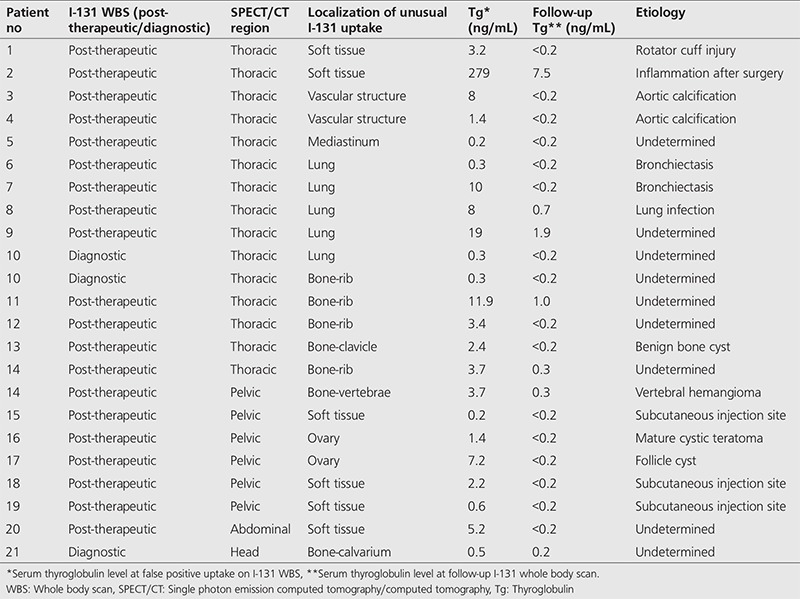
Localization sites and possible etiologies of false positive I-131 uptakes
